# Frequency of EBV associated classical Hodgkin lymphoma decreases over a 54-year period in a Brazilian population

**DOI:** 10.1038/s41598-018-20133-6

**Published:** 2018-01-30

**Authors:** Antonio Hugo Jose Froes Marques Campos, Adriana Moreira, Karina Braga Ribeiro, Roberto Pinto Paes, Maria Claudia Zerbini, Vera Aldred, Carmino Antonio de Souza, Cristovam Scapulatempo Neto, Fernando Augusto Soares, Jose Vassallo

**Affiliations:** 1 Department of Anatomic Pathology, A. C. Camargo Cancer Center, São Paulo, Brazil; 2 Department of Collective Health, Irmandade da Santa Casa de Misericordia de São Paulo, São Paulo, Brazil; 3 Department of Anatomic Pathology, Irmandade da Santa Casa de Misericordia de São Paulo, São Paulo, Brazil; 4 Department of Anatomic Pathology, Faculdade de Medicina da Universidade de São Paulo, São Paulo, Brazil; 5 Hematology Clinics, Faculdade de Ciências Médicas da Universidade Estadual de Campinas (Unicamp), Campinas, São Paulo, Brazil; 6 Department of Anatomic Pathology, Hospital do Câncer de Barretos, São Paulo, Brazil; 7 Laboratory of Investigative Pathology, CIPED, Faculdade de Ciências Médicas da Universidade Estadual de Campinas (Unicamp), Campinas, São Paulo, Brazil; 8 Present Address: Pathology - D’Or Hospitals Network e Faculty of Dentristry, USP, São Paulo, Brazil; 9 Present Address: DASA - Diagnósticos das Américas, Barueri-SP, Brazil

## Abstract

The epidemiology of classical Hodgkin lymphoma varies significantly in populations with different socioeconomic conditions. Among other changes, improvement in such conditions leads to a reduction in the association with EBV infection and predominance of the nodular sclerosis subtype. This study provides an overview of the epidemiology of 817 cases of classical Hodgkin lymphoma diagnosed in five reference hospitals of the State of Sao Paulo, Brazil, over 54 years (1954–2008). The cases were distributed in 3 periods (1954–1979; 1980–1999; and 2000–2008). EBV-positive cases decreased from 87% to 46%. In children and adolescents (<15 years) and in young adults (15–45 years), EBV-positive cases decreased respectively from 96% to 64%, and from 85% to 32%. The percentage of male patients declined from 80% to 58%. In older patients (>45 years), the decrease in EBV infection was not significant. Nodular Sclerosis was the most common subtype in all periods. These results support the hypothesis that, in the Brazilian State of Sao Paulo, classical Hodgkin lymphoma has changed and now shows characteristics consistent with Pattern III observed in populations that experienced a similar socioeconomic transition.

## Introduction

Epidemiological studies suggest that classical Hodgkin lymphoma (cHL) develops in patients previously exposed to Epstein-Barr virus (EBV), which infects more than 90% of individuals who reach adulthood. The onset of EBV-positive cHL in children may be due to an aberrant response to an early EBV infection, whereas the decline in EBV-specific immunity (with loss of control over latent infection) may be responsible for the occurrence of EBV-positive cHL in older adults^1^. In developed nations, the epidemiology of cHL shows a distinct clinical and pathological pattern, characterized by a peak of incidence in young adults, predominance of Nodular Sclerosis (NS) subtype and low association with Epstein-Barr virus (EBV) infection. According to previous studies, this is the last of three identified patterns seen in this disease^2,3^. Pattern I is usually seen in populations with low socioeconomic status, and is characterized by a peak of incidence in childhood, predominance of Mixed Cellularity (MC) subtype and high association with EBV. Populations experiencing a transition in their socioeconomic status show an intermediate pattern (Pattern II). A recent report has shown that, in the Republic of Korea, a shift from pattern II to pattern III has occurred over a period of 31 years, reflecting the impact of social and economic development in this country^4^. Similar studies in other countries, however, have identified a mixed pattern (Pattern II with a bias to Pattern I or Pattern III, depending on the influence of more pronounced poorer or improved socioeconomic conditions)^5–12^.

For the past 3 decades, Brazil has also experienced a marked socioeconomic transition. It is expected that the epidemiology of cHL during this transition might have changed, as reported in other populations. Since no previous studies have tried to examine temporal trends of cHL in the Brazilian population, we sought to undertake this task by examining 817 cases of cHL diagnosed and treated in the State of Sao Paulo during 1954 and 2008.

## Results

### Main clinicopathological features of the total group

The clinical and pathological features of the 817 cases are listed on Table 1. The median age at diagnosis for the whole group was 27 years (range 2–91 years). The male to female (MF) ratio was 1.62:1. NS was the predominant subtype (70% of all cases), followed by MC, Lymphocyte Rich (LR) and Lymphocyte Depleted (LD) subtypes (23%, 2% and 2% of all cases, respectively). Those classified as “not otherwise specified” accounted for 3% of all cases. Most patients (54%) were classified as stage II or III by Ann Arbor criteria. Approximately 56% of the cases were EBV-positive. Detection of EBV by immunohistochemistry (LMP1) was positive in 46.5% of the cases. The EBER-ISH technique showed that 49.5% of the cases were positive. In 39.7% of the cases we observed agreement between immunohistochemistry and “*in situ*” hybridization. A substantial agreement between the two methods was observed (κ = 0.649). The finding of LMP1-positive/EBER-negative cases (n = 55) was probably due to technical problems, mainly destruction of cytomorphological features that did not benefit from adjustments of Proteinase K concentration or incubation time. Most LMP1-positive/EBER-negative cases (71.8%) belonged to the period between 1954 and 1999.

**Table 1:** Clinico-pathological features of 817 patients diagnosed with classical Hodgkin lymphoma.

Parameter	N	%	1954–1979 (n = 128)	%	1980–1999 (n = 320)	%	2000–2008 (n = 369)	%	*P*
EBV status									
Positive	459	56	111	87	179	56	169	46	<0.0001*
Negative	358	44	17	13	141	44	200	54	
Age (years)									
<15	155	19	26	20	76	24	53	14	0.02
15–44	483	59	73	57	185	58	225	61	
≥45	179	22	29	23	59	18	91	25	
Gender									
Male	506	62	103	80	189	59	214	58	0.0001*
Female	311	38	25	20	131	41	155	42	
Histology									
Nodular sclerosis	572	70	79	62	222	69	271	73	0.005
Mixed celularity	185	23	33	26	78	24	74	20	
Lymphocyte rich	17	2	1	1	5	2	11	3	
Lymphocyte depletion	18	2	7	5	8	3	3	1	
Unclassifiable	25	3	8	6	7	2	10	3	
Ann Arbor Staging									
I-II	283	35	26	20	104	33	152	41	0.002*‡
III-IV	356	44	69	54	121	38	167	45	
NA	178	21	33	26	95	30	50	14	

*Cochran-Armitage test for trend. ^‡^Analysis included only Ann-Arbor stages I-II and III-IV. NA: Data not available.

The Pearson chi-squared test was used to assess for the presence of an association between variables with more than two categories (age and gender) and time periods.

### Trends in age and gender distribution

Supplemental Figure 1 shows the age distribution of cHL over time. When we stratified the analysis by age groups (Table 1), we observed an increase in the proportion of patients between 15–45 years and a decrease in the proportion of children and adolescents (<15 years), while the proportion of patients over 45 years remained relatively stable (P = 0.02).

**Figure 1:**
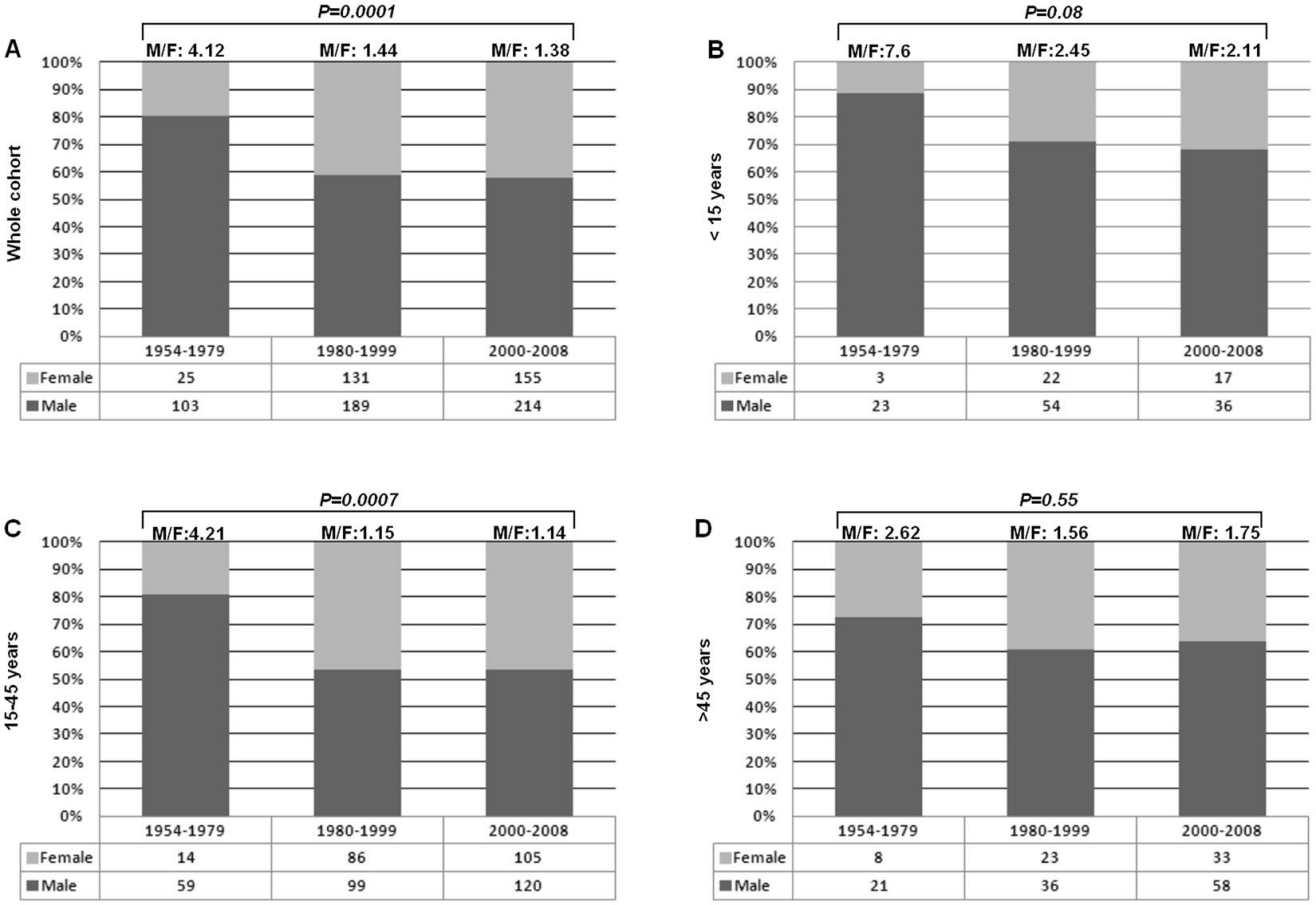
Gender distribution of patients diagnosed with classical Hodgkin lymphoma over 54 years. (**A**) Whole cohort; (**B**) Patients under 15 years; (**C**) Patients between 15–44 years; (**D**) Patients aged 45 years and older. Cochran-Armitage test for trend.

The proportion of male patients declined from 80% to 58% (P < 0.001, Fig. 1A and Table 1). In children and adolescents (<15 years), although the percentage of male patients decreased from 88% in the first period (1954–1979) to 68% in the third period (1980–1999), this change was not statistically significant (P = 0.08).

On the other hand, a significant change was observed in the group of young adults (15–45 years). There was a significant decrease in the percentage of male patients between the first and third periods (from 81% to 53%, P < 0.001).

Finally, in the group over 45 years of age, the percentage of male patients decreased from 72% in the first period to 64% in the third period, although this change was not statistically significant (P = 0.55).

### Trends in cHL subtype distribution

As described earlier, NS was the predominant subtype (accounting for 70% of all cases), followed by MC subtype (23%). Interestingly, NS was the predominant subtype in all periods (62% in the first period; 69% in the second period; and 73% in the third period). We observed a significant change in the proportions of the histological subtypes (Table 1, P = 0.005). However, this change was due to an increase in the proportion of LR subtype (from 1% to 3%) and, most importantly, to a decrease in the proportion of LD subtype (from 5% to 1%). The proportions of NS and MC subtypes did not vary significantly.

### Trends in cHL stage at diagnosis

Table 1 shows the proportion of cases over time according to Ann Arbor staging. There has been an increase in the diagnosis of early stage cases over the three periods studied (P = 0.002).

### Time-trends in EBV prevalence

**Figure 2:**
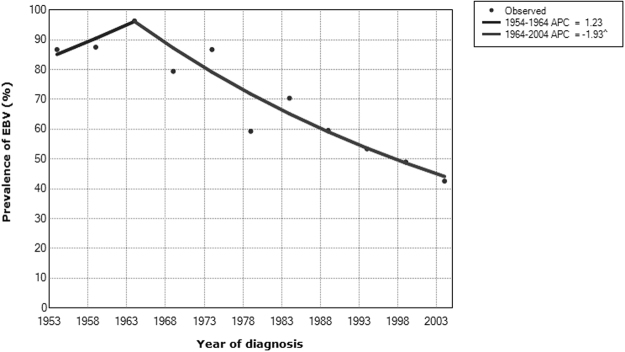
Prevalence of EBV-positive classical Hodgkin lymphoma over 54 years. Jointpoint trend analysis.

**Figure 3:**
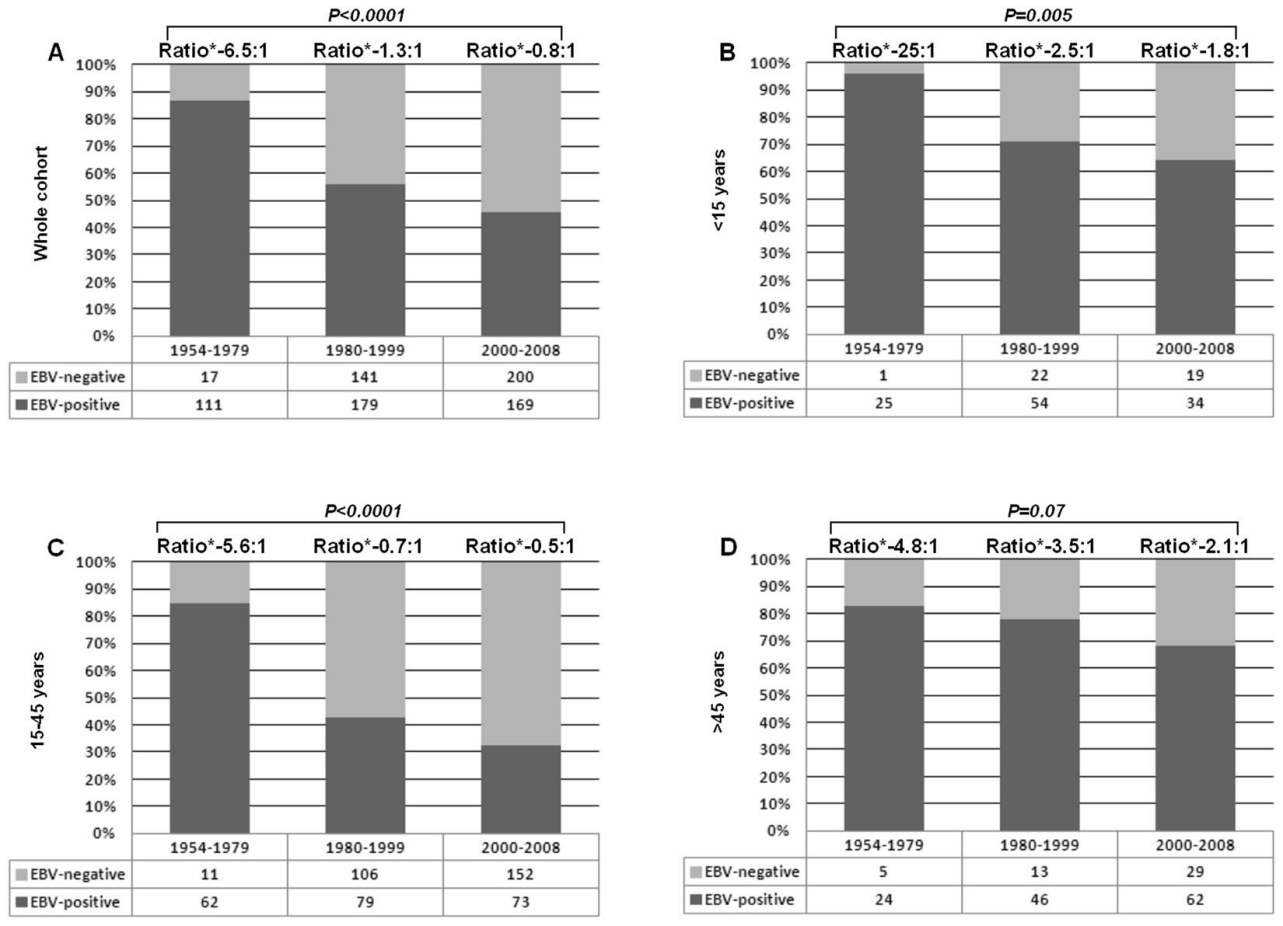
Frequency of EBV-associated classical Hodgkin lymphoma over 54 years. (**A**) Whole cohort; (**B**) Patients under 15 years; (**C**) Patients between 15–44 years; (**D**) Patients aged 45 years and older. Cochran-Armitage test for trend.

After an initial increase in the prevalence of EBV + tumors in the period 1954–1968 (increase of 1.23% every five years), we saw a significant decline from 1969, with a reduction of 1.93% every five years (95% CI−2.47,−1.38, Fig. 2). When analyzed by periods, we saw that EBV-positivity decreased from 87% in the first period to 46% in the third period (P < 0.0001, Fig. 3A and Table 1). Data were also analyzed by age groups (Fig. 3B). In children and adolescents (<15 years), EBV-positivity decreased from 96% in the first period to 64% in the third period (P = 0.005).

The most impressive change was observed among young adults (15–45 years), in which EBV-positivity decreased by more than 50% (from 85% in the first period to 32% in the third period; P < 0.001).

On the other hand, in the group over 45 years of age, there was no statistically significant change in percentage of EBV-positive cases; P = 0.07).

**Figure 4:**
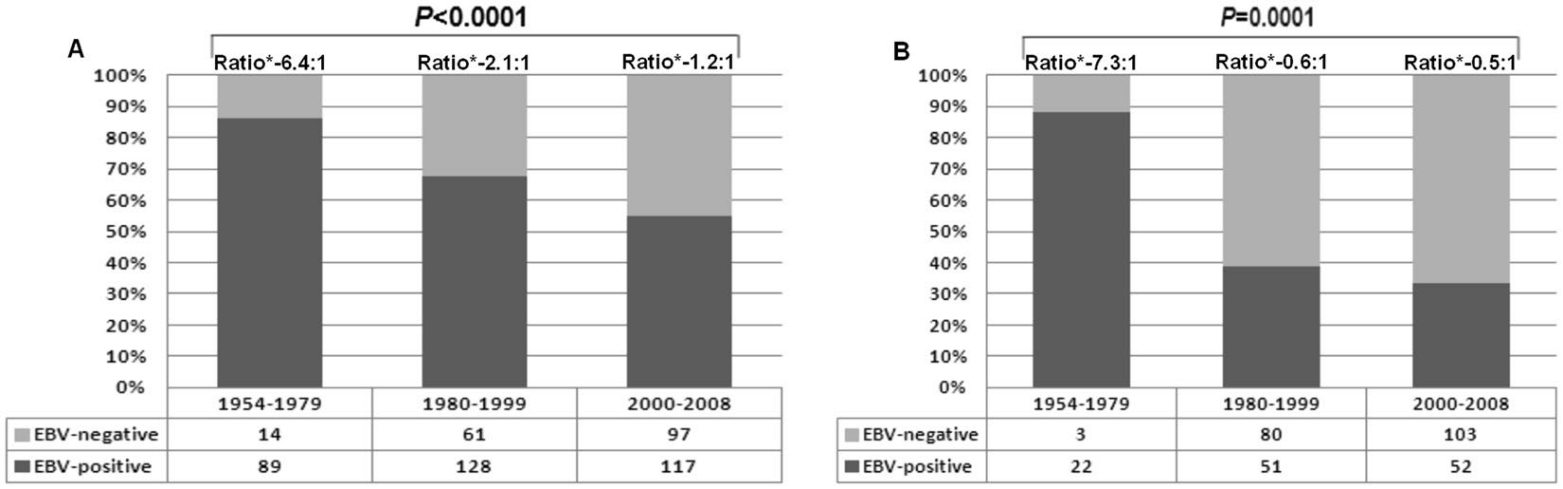
Frequency of EBV-associated classical Hodgkin lymphoma over 54 years, according to gender (**A**) males; (**B**) Females. Cochran-Armitage test for trend.

Overall, EBV infection was more common among male patients (with an EBV ratio of 2:1 compared to an EBV ratio of 0.7:1 in female patients). When considered by periods, we observed that in males, there was a sharp decrease in the percentage of EBV-positive cases (from 86% in the first period to 55% in the third period, P < 0.0001) (Fig. 4A). In females, the percentage of EBV-positive cases declined by more than 50% (from 88% in the first period to 33% in the third period, P = 0.0001) (Fig. 4B).

**Figure 5:**
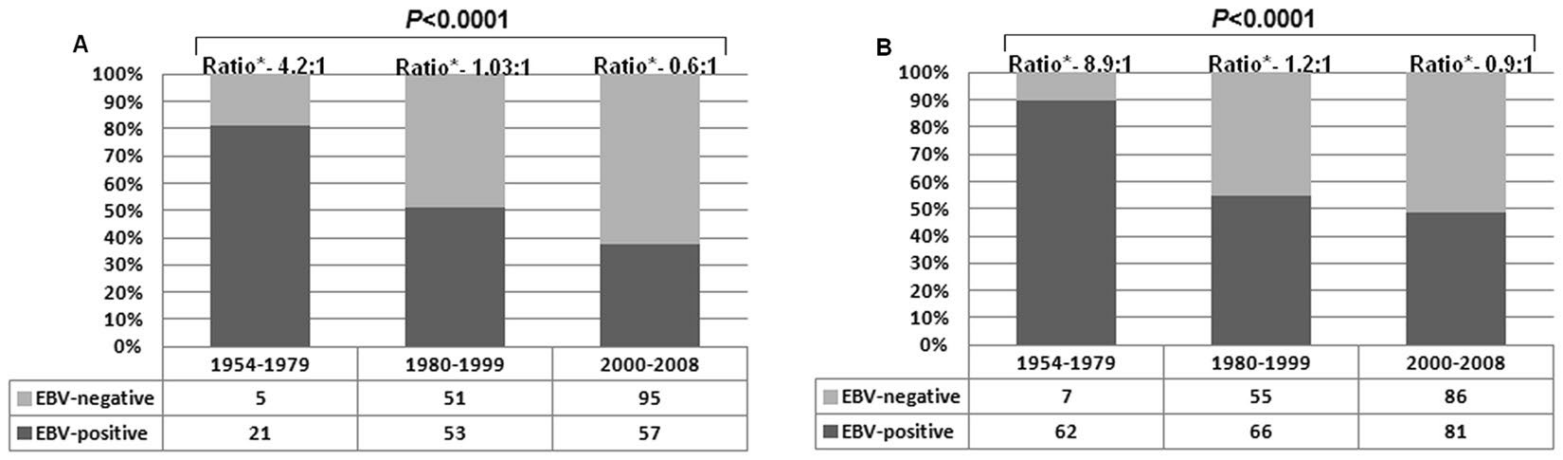
Frequency of EBV-associated classical Hodgkni lymphoma over 54 years, according to Ann Arbor Stage ((**A**)-Stage I-II; (**B**)-Stage III-IV). Cochran-Armitage test for trend.

We also observed a change in the percentage of EBV infection according to clinical staging. In early stage patients (Ann Arbor I-II, Fig. 5A), the percentage of EBV-positive cases dropped from 81% in the first period to 37% in the third period (P < 0.0001). In late stage patients (Ann Arbor III-IV, Fig. 5B), it dropped from 90% to 48% (P < 0.001).

**Figure 6:**
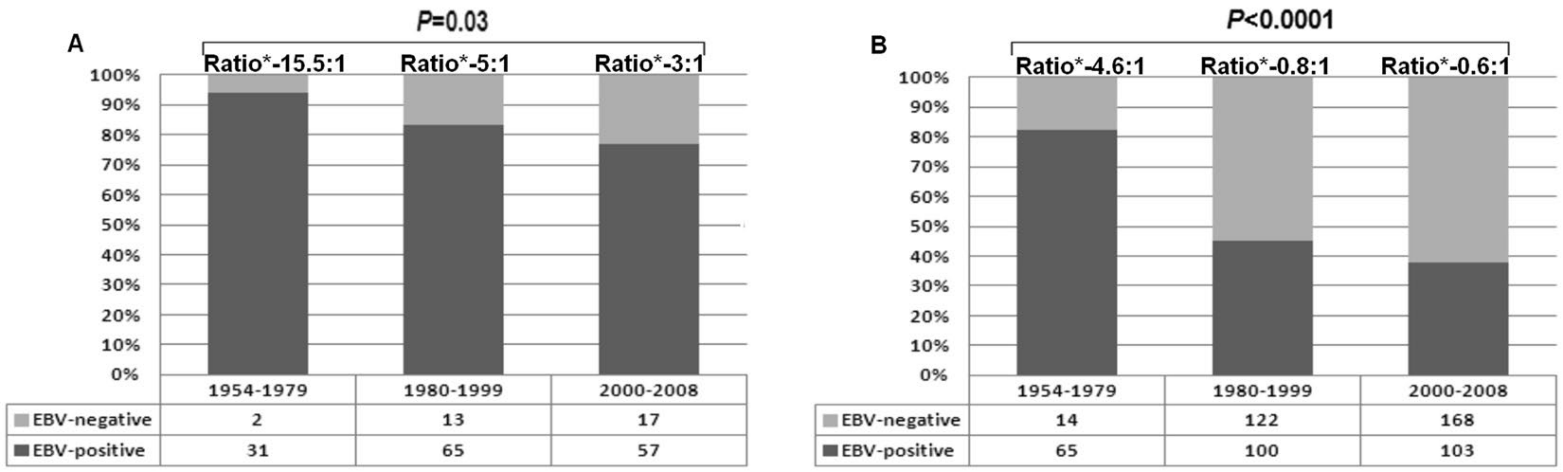
Frequency of EBV-associated classical Hodgkin lymphoma over 54 years, according to histological type (**A**) mixed cellulatiry; (**B**) nodular sclerosis. Cochran-Armitage test for trend.

Finally, we observed a temporal change in the rate of EBV infection according to histological type. In the first period, most MC and NS cases were frequently associated with EBV infection. In the second and third periods, however, MC cases were more likely to be EBV-positive than NS cases (Fig. 6). Between the first and third periods, the percentage of EBV-positive cases in MC cHL dropped from 94% to 77% (P = 0.03), while in NS cHL it dropped from 82% to 38% (P < 0.001).

## Discussion

Previous studies (including reports by some of the authors of this work) tried to address the epidemiology of cHL in the Brazilian population (Supplemental Table S1)^13–19^. However, this is the first study to assess long-term temporal trends of cHL in a large series of Brazilian patients, although with the limitation of being a hospital-based study. Our analysis revealed that, over 54 years, the epidemiology of cHL has changed and now shows features consistent with Pattern III described in developed countries (pronounced initial peak in young adults, lower rates of EBV-associated infection, and predominance of the NS subtype)^3^.

The most striking finding is the sharp global decrease in EBV-positive cases. The frequency observed in the third period (46% of the cases diagnosed between 2000–2008) is similar to that observed in developed countries^20^. In children and adolescents (<15 years), the frequency of EBV-positive cHL decreased approximately 14 times. Among young adults (15–45 years), there was an inversion in the EBV ratio, which equals to a decrease in frequency of approximately 12 times. In these two groups, the decrease in the frequency of EBV-positive cHL can be explained by improvements in socioeconomic conditions over the last 3 decades. Only among patients older than 45 years of age there was a small decrease (2-fold) in the frequency of EBV infection. The persistent higher proportion of EBV-positive cases in this group can be explained by progressively reduced immunosurveillance, which may result in viral reactivation^20^.

When time trends in EBV-frequency were analyzed by gender, we observed that even in males, who usually have higher rates of EBV-associated cHL than females, the frequency of EBV-infection dropped 5 times. In female patients, the frequency of EBV-associated cHL decreased approximately 15 times. Although the underlying cause for higher rates of EBV-associated cHL in males is currently undetermined, the literature suggests that this association is due to a diminished cellular immunity compared to female patients^21,22^. Low socioeconomic conditions may explain why, in the first period, women and men had a rather similar rate of EBV-associated cHL (as this fact would undermine a relative female advantage in terms of effectiveness of the immune system). Likewise, the improvement in socioeconomic conditions over the years may explain why women performed 3 times better than men in diminishing the rate of EBV-associated cHL. Improved socioeconomic conditions may also explain why the rate of EBV-infection plummeted both in early (Ann-Arbor I-II) and late (III-IV) stage patients.

Other findings consistent with Pattern III are the predominance of NS subtype of cHL and an age distribution with a peak at the third decade. As observed by other studies (Supplementary Table S1), the NS subtype was the commonest in all the three periods.

Additional findings of interest were the decline in the global MF ratio (which was due mainly to the decline in the MF ratio among young adults), the significant increase in the proportion of LR subtype and the decrease in LD subtype, and the increase in the diagnosis of early stage cases.

Our study also found that the decline in the rate of EBV infection was not so drastic in the MC subtype (as compared to the NS subtype) or in the group of patients aged over 45 years of age. In the former case, the biological basis for the persistent association of EBV infection with the MC subtype is currently unknown. In the latter case, this finding could be explained by the viral reactivation secondary to ineffective immunosurveillance^20^.

Overall, our findings parallel those observed in other countries that experienced a similar socioeconomic transition in the recent decades. In a hospital-based study of 385 cases of cHL in South Korea between 1980 and 2011, Koh *et al*. have also observed a decrease in the frequency of association with EBV, most pronounced in children and young adults^4^. EBV-positive cHL also decreased in males, females and in all clinical stages. The authors also observed a decrease in the proportion of children diagnosed with cHL, as well as in the proportion of LD histological subtype.

Another interesting study for comparison was undertaken by Huang *et al*., in which the epidemiology of cHL in Northern China was evaluated in relation to that of the Netherlands^11^. The age distribution in our study is similar to that of Northern China, a finding also reported in Taiwan^23^.

The present study, however, has the same limitations of other hospital-based studies. Although the major advantages of such studies are the possibility of case reclassification and the assessment of EBV by immunohistochemistry and/or “*in situ*” hybridization, caution must be exercised when extrapolating data to the general population. This is particularly true in Brazil, a country in which disparities in socioeconomic conditions are observed among the different regions. Our study assessed cases diagnosed in major tertiary hospitals in the state of Sao Paulo, which is the wealthiest and more developed of all states in Brazil. However, this cohort is small compared to the annual estimate of new cases in the state of Sao Paulo (520 new cases diagnosed in 2016) or in the country (2470 cases in the same year)^24^, and due to variation in availability and completeness of data about cHL over time in Brazil, it is not possible to assess whether other factors (besides exposure to EBV) may have influenced the decrease in the proportion of EBV-positive cases^25^. Additionally, our data reflect solely the percentage of cases by age and not age-specific incidence rates of the disease (in fact, available published data from population-based cancer registries show that, in Brazil, age-specific incidence rates seem to follow a bimodal distribution usually seen in resource-rich countries)^26–28^.

Finally, the size of the groups in the 3 different age categories may have impacted our results, since the oldest group has much less patients than the second and third groups. This may explain the apparent transient initial increase in the incidence of EBV-positive Hodgkin lymphoma (Fig. 2**)** and was probably due to the lack of availability of old tissue blocks or referral patterns.

Nevertheless, we believe that our findings represent the general population in this state because some of them are in accordance with data from the Fundação Oncocentro de São Paulo (FOSP, a public state institution that records cancer cases and undertakes epidemiological studies). Data available from 2000 to 2013 show that there were 4943 cases of Hodgkin lymphoma reported from 77 hospitals to the cancer registry, with a MF ratio of 1.2:1 (our data shows a MF ratio of 1.38:1 for cases diagnosed between 2000 and 2008). Additionally, the age distribution of the cases recorded by FOSP is similar to that observed in our study^29^.

However, the Pattern III that we identified in this population might not have been reached by other states, in which social inequality is more marked. Although all institutions are reference centers that receive patients from other parts of the country, this study cannot address this question adequately using the information available in the medical records, since those patients that were born in other states/regions but lived in the State of São Paulo by the time of their diagnosis cannot by reliably distinguished from those that lived in other states/regions and came to the institutions only for medical attention. It is not also possible to entirely exclude the possibility that the pattern of cHL cases have changed as a consequence of changing patterns of referral to the hospitals included in this study. To address this hypothesis, a broader study including institutions from all regions of the country would be desirable.

In conclusion, we have found that the epidemiology of cHL in the Brazilian state of Sao Paulo has reached Pattern III, particularly in terms of EBV association and age distribution, as already seen in other countries that experienced a similar socioeconomic transition. Our study also shows that the impact of the socioeconomic improvement in the rate of EBV infection is not observed in older patients, or in the MC type, as the interaction of the disease with the immune system may overcome other potential pathogenetic factors.

## Materials and Methods

### Ethics approval

This was a collaborative study conducted in five reference hospitals located in the State of São Paulo, Brazil (A. C. Camargo Cancer Center, Hospital de Clínicas da Faculdade de Medicina da Universidade de São Paulo (HC-FMUSP), Hospital Central da Irmandade da Santa Casa de Misericórdia de São Paulo (ISCMSP), Hospital de Clínicas da Faculdade de Ciências Médicas da Universidade Estadual de Campinas (UNICAMP) and Hospital do Câncer Pio XII de Barretos. Independent approval was obtained from the Ethics Review Board (ERB) of A C Camargo Cancer Center (approval number 1120/08), according to relevant guidelines and regulations. Informed consent was given by the patients for the use of their tissue samples, as well as the use of associated clinical and pathological information. For minors/children or legally incapacitated patients, written consent was obtained from the next of kin, caretakers, or guardians. When specific written consent was not possible to obtain prospectively, the Ethics Review Board authorized the use of samples and associated data, according to national guidelines. The data was analyzed anonymously.

### Case selection and clinical-pathological review

Cases diagnosed from 1954 to 2008 as Hodgkin lymphoma/disease, Hodgkin granuloma, Hodgkin sarcoma, Hodgkin paragranuloma, malignant lymphogranuloma, malignant granuloma, lymphosarcoma and reticulosarcoma were retrieved from the pathological records of the collaborating institutions. Conventional and immunohistochemical stains were reviewed and classified by three pathologists (JV, FAS, AHC), according to the criteria of the WHO Classification of Tumors of Haematopoietic and Lymphoid Tissues^28^. Additional immunoistochemical analysis was performed in old or doubtful cases. Clinical data were collected from medical charts and included age, gender, and Ann Arbor Staging. Because the clinical data at the three institutions were incomplete or variable over time, information did not include presence of B symptoms, bulky disease and, for patients aged over 15 years, the International Performance Status (IPS)^30^. Samples lacking sufficient clinical information, formalin-fixed and paraffin-embedded tissue or relapse biopsies were excluded. Cases associated with HIV-infection were also excluded.

From the initial 863 cases, 46 patients were excluded: 14 were associated with HIV-infection, 13 had insufficient tissue for the tests for EBV status, and 19 had insufficient clinical data. The 817 remaining cases were: 312 from the A C Camargo Cancer Center, 202 from the ISCMSP, 148 from the HC-FMUSP, 110 from Hospital do Cancer Pio XII, and 45 cases from UNICAMP.

Cases were also classified according to the era of diagnosis: 1954 to 1979 (128 cases), 1980 to 1999 (320 cases), and 2000 to 2008 (369 cases). Time intervals were chosen to reflect the consistent changes in the economic, demographic and epidemiological indicators observed in Brazilian demographic censuses conducted by the Brazilian Institute of Geography and Statistics (IBGE)^31^. For statistical purposes concerning age, three groups were characterized: younger than 15 years (children/adolescents), 15–45 years, and older than 45 years.

### Immunohistochemistry and “*in situ*” hybridization for EBV

Tissue microarrays (TMAs) and immunohistochemistry were done at the A. C. Camargo Cancer Center. The TMAs were built as reported elsewhere^32^. Each case was spotted in duplicate. Immunohistochemistry was performed manually, with a primary antibody to the EBV latent membrane protein-1 (LMP-1, clone CS1–4, 1:100 dilution, Novocastra, Newcastle upon Tyne, UK), as previously described^33^. Cases were also tested for the presence of EBV RNAs using an “*in situ*” hybridization (ISH) kit (EBER oligoprobe, Novocastra). A previously known positive case of cHL was used as an external positive control. Negative controls were also used in each run, by omitting the primary antibodies on the same case used as positive control.

After staining, slides were evaluated by the three hematopathologists (JV, FAS, AHC), that were blinded to case details such as age, gender, stage, or previous EBV status. For divergent results, a consensus was obtained by all three pathologists, using a multihead microscope. Only classical diagnostic Reed-Sternberg cells, Hodgkin cells or variants with undisputable morphology were considered. The cases that generated disagreement (11.2%; n = 91) were mainly due to the differentiation between mixed cellularity or nodular sclerosis and frequently lacked sufficient tissue to allow proper classification. In 497 cases (60.8%) the original diagnosis was confirmed. This was expected, since a substantial number of cases were classified with previous classification schemes. In 320 cases (39.2%), the original diagnosis was partially modified; in 181 of these cases (belonging to the first and second periods), this modification resulted from the update of the nomenclature used at diagnosis. In the remaining cases (n = 139) it was due to the update of the histological subtype.

EBV was considered positive or negative for LMP1 and/or EBER following the recommendations by Gulley *et al*.^34^. Agreement between the two methods was analyzed using Cohen’s kappa. The strength of agreement of the kappa statistic was interpreted as follows: <0.00 = poor agreement, 0.00–0.20 = slight agreement, 0.21–0.40 = fair agreement, 0.41–0.60 = moderate agreement, 0.61–0.80 = substantial agreement, 0.81–1.00 = almost perfect agreement^35^.

### Statistical analysis

Statistical analyses were performed using the statistical package Graph Pad Prism (version 5.02, GraphPad Software Inc., San Diego, CA, USA) or MedCalc software (version 11.0.1, MedCalc Software, Belgium). The Cochran-Armitage test was used to assess whether a changing trend in the disease pattern has occurred analyzing the 3 periods: 1954–1979; 1980–1999; and 2000–2008. Pearson chi-square or Fisher’s exact tests were used to evaluate the association between categorical variables, while Student *t* test was used to compare means from independent samples. Time-trends in EBV prevalence were also assessed using Jointpoint regression. Results were considered statistically significant when *p* < 0.05.

## Electronic supplementary material

Supplementary Information

## References

[1] Grywalska E, Rolinski J (2015). Epstein-Barr virus-associated lymphomas. Semin Oncol.

[2] Correa P, O’Conor GT (1971). Epidemiologic patterns of Hodgkin’s disease. Int J Cancer.

[3] Glaser SL, Jarrett RF (1996). The epidemiology of Hodgkin’s disease. Bailliere Clin Haematol.

[4] Koh YW (2013). Changing trend of Epstein-Barr virus association in Hodgkin lymphoma in the Republic of Korea. Ann Hematol.

[5] Zarate-Osorno A, Roman LN, Kingma DW, Meneses-Garcia A, Jaffe ES (1995). Hodgkin’s disease in Mexico. Prevalence of Epstein-Barr virus sequences and correlations with histologic subtype. Cancer.

[6] Chang KL, Albújar PF, Chen YY (1993). High prevalence of Epstein-Barr virus in the Reed-Sternberg cells of Hodgkin’s disease occurring in Peru. Blood.

[7] Leoncini L (1996). Neoplastic cells of Hodgkin’s disease show differences in EBV expression between Kenya and Italy. Int J Cancer.

[8] Makar RR, Saji T, Junaid TA (2003). Epstein-Barr virus expression in Hodgkin’s lymphoma in Kuwait. Pathol Oncol Res.

[9] Al-Salam S, John A, Daoud S, Chong SM, Castella A (2008). Expression of Epstein-Barr virus in Hodgkin lymphoma in a population of United Arab Emirates nationals. Leuk Lymphoma.

[10] Hjalgrim H, Seow A, Rostgaard K, Friborg J (2008). Changing patterns of Hodgkin lymphoma incidence in Singapore. Int J Cancer.

[11] Huang X (2011). Epidemiology of classical Hodgkin lymphoma and its association with Epstein Barr virus in Northern China. PLoS One.

[12] Sughayer MA, Haddad HA, Al-Yousef RM, El-Khateeb M, Abu-Rass H (2014). Epstein-Barr virus and Hodgkin lymphoma in Jordan. Hematol Oncol Stem Cell Ther.

[13] de Souza CA, Vassallo J, Lorand-Metze I (1997). Hodgkin’s disease in Brazil: a clinicopathologic study. Haematologica.

[14] Vassallo J, Metze K, Traina F, de Souza CA, Lorand-Metze I (2001). Expression of Epstein-Barr virus in classical Hodgkin’s lymphomas in Brazilian adult patients. Haematologica.

[15] Elgui de Oliveira D, Bacchi MM, Abreu ES, Niero-Melo L, Bacchi CE (2002). Hodgkin disease in adult and juvenile groups from two different geographic regions in Brazil: characterization of clinicopathologic aspects and relationship with Epstein-Barr virus infection. Am J Clin Pathol.

[16] Vassallo J (2005). Histological classification of 1,025 cases of Hodgkin’s lymphoma from the State of São Paulo, Brazil. Sao Paulo Med J..

[17] Araujo I (2006). The high frequency of EBV infection in pediatric Hodgkin lymphoma is related to the classical type in Bahia, Brazil. Virchows Arch.

[18] Chabay PA (2008). Pediatric Hodgkin lymphoma in 2 South American series: a distinctive epidemiologic pattern and lack of association of Epstein-Barr virus with clinical outcome. J Pediatr Hematol Oncol.

[19] Barros MH, Hassan R, Niedobitek G (2011). Disease patterns in pediatric classical Hodgkin lymphoma: a report from a developing area in Brazil. Hematol Oncol.

[20] Kapatai G, Murray P (2007). Contribution of the Epstein Barr virus to the molecular pathogenesis of Hodgkin lymphoma. J Clin Pathol.

[21] Chang ET (2004). Heterogeneity of risk factors and antibody profiles in epstein-barr virus genome-positive and -negative hodgkin lymphoma. J Infect Dis.

[22] Whitacre CC, Reingold SC, O’Looney PA (1999). A gender gap in autoimmunity. Science.

[23] Lee MY, Tan TD, Feng AC (2005). Clinico-pathological study of Hodgkin’s lymphoma in a cancer center in Taiwan. Clin Lab Haematol.

[24] Instituto Nacional do Câncer. Available from, http://www.inca.gov.br/estimativa/2016/. Acessed 7 November 2017.

[25] Kusminsky G, Abriata G, Forman D, Sierra MS (2016). Hodgkin lymphoma burden in Central and South America. Cancer Epidemiol 2016.

[26] International Agency for Research on Cancer. Cancer Incidence in Five Continents [Internet]. Availavle from, http://ci5.iarc.fr. Accessed 27 December 2017.

[27] Ferlay J *et al*. GLOBOCAN 2012 v1.0, Cancer Incidence and Mortality Worldwide: IARC CancerBase No. 11 [Internet]. Lyon, France: International Agency for Research on Cancer; Available from: http://globocan.iarc.fr. Accessed 27 December 2017 (2013)

[28] Swerdlow, S. H. *et al*. *WHO Classification of Tumors: Pathology and Genetics of Tumors of Haematopoietic and Lymphoid Tissues*. 4^th^ ed (eds Bosman, F. T., Jaffe, E. S., Lakhani, S. R. & Ohgaki, H.) 321–334 (Lyon, France: International Agency for Research on Cancer, 2008).

[29] Fundação Oncocentro de São Paulo. Available from www.fosp.saude.sp.gov.br/publicacoes/rhc. Accessed 21 July 2017.

[30] Hasenclever D, Diehl V (1998). A prognostic score for advanced Hodgkin’s disease. International Prognostic Factors Project on Advanced Hodgkin’s Disease. N Engl J Med.

[31] Instituto Brasileiro de Geografia e Estatística (IBGE). Indicadores Sociodemográficos e de Saúde no Brasil. Available from, https://www.ibge.gov.br/estatisticas-novoportal/sociais/saude/9336-indicadores-sociodemograficos-e-de-saude-no-brasil.html. Acessed 7 November 2017.

[32] Campos AH, Vassallo J, Soares FA (2013). Matrix metalloproteinase-9 expression by Hodgkin-Reed-Sternberg cells is associated with reduced overall survival in young adult patients with classical Hodgkin lymphoma. PLoS One.

[33] Campos AH, Aldred VL, Ribeiro KC, Vassallo J, Soares FA (2009). Role of immunoexpression of nitric oxide synthases by Hodgkin and Reed-Sternberg cells on apoptosis deregulation and on clinical outcome of classical Hodgkin lymphoma. Mol Cell Biochem.

[34] Gulley ML (2002). Guidelines for interpreting EBER *in situ* hybridization and LMP1 immunohistochemical tests for detecting Epstein-Barr virus in Hodgkin Lymphoma. Am J Clin Pathol.

[35] Landis JR, Koch GG (1977). The measurement of observer agreement for categorical data. Biometrics.

