# Supplementary material for: Frequency of EBV associated classical Hodgkin lymphoma decreases over a 54-year period in a Brazilian population
Source: Sci Rep. 2018 Jan 30;8:1849. doi: 10.1038/s41598-018-20133-6 (PMC5789833; doi:10.1038/s41598-018-20133-6)
Supplement: Supplementary file 1 — Supplementary Information [file 41598_2018_20133_MOESM1_ESM.pdf]

Frequency of EBV associated classical Hodgkin lymphoma decreases over a 54-year period in a Brazilian population

Antonio Hugo Jose Froes Marques Campos[1,a,*]
Adriana Moreira [1,a]
Karina Braga Ribeiro [2]
Roberto Pinto Paes [3]
Maria Claudia Zerbini [4]
Vera Aldred [3]
Carmino Antonio de Souza [5]
Cristovam Scapulatempo Neto [6]
Fernando Augusto Soares [1]
Jose Vassallo [1,7]

[1] Department of Anatomic Pathology, A. C. Camargo Cancer Center, São Paulo, Brazil
[2] Department of Collective Health, and [3] Department of Anatomic Pathology, Irmandade da Santa Casa de Misericordia de São Paulo, São Paulo, Brazil
[4] Department of Anatomic Pathology, Faculdade de Medicina da Universidade de São Paulo, São Paulo, Brazil
[5] Hematology Clinics, and [7] Laboratory of Investigative Pathology, CIPED, Faculdade de Ciências Médicas da Universidade Estadual de Campinas (Unicamp), Campinas, São Paulo, Brasil
[6] Department of Anatomic Pathology, Hospital do Câncer de Barretos, São Paulo, Brazil
[a] Both authors contributed equally to this work.

<u>* Correspondence to</u>:
Antonio Hugo J. F. M. Campos, M.D.
Department of Anatomic Pathology, A C Camargo Cancer Center

e-mail: ahcampos@accamargo.org.br

Supplemental information:

Figure S1: Age distribution of cHL over time.

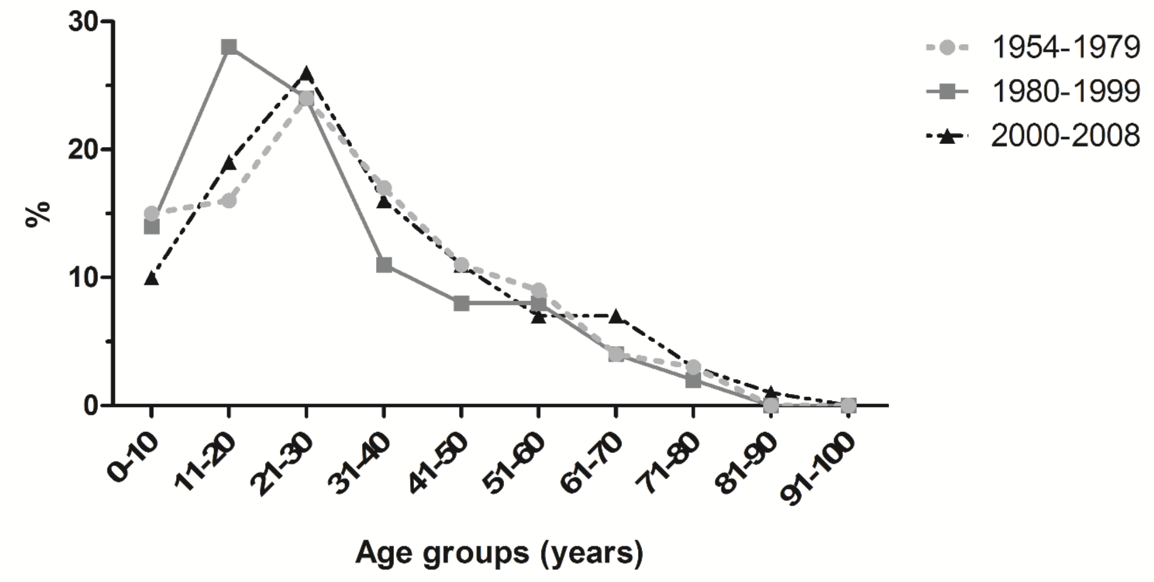

Supplemental table S1: Previous studies on classical Hodgkin Lymphoma in the Brazilian population

Author	Year	Study population and main findings	Reference
de Souza CA, et al.	1997	Number of patients: 134. Date range (diagnosis): 1985-1994. Patients above 15 years (age range not provided). Male to female ratio: 1.31/1. NS was the predominant subtype, followed by MC. Incidence peak between 21-30 years, followed by a descending curve. EBV testing: not performed.	13
Vassallo J, et al.	2001	Number of patients: 78. Date range (diagnosis): 1987-1998. Patients above 15 years (age range, 15-75 years). NS cases predominant. EBV detected in 64.1% of the cases, more common in the MC subtype.	14
Elgui de Oliveira D, et al.	2002	Number of patients: 96. Date range (diagnosis): not provided. Pediatric (<20 years) and adult (20 years or older) cases. Forty-eight percent of the cases diganosed in the State of São Paulo (age range, 2-70 years). Fifty-two percent of the cases diagnosed in the Northeastern State of Ceará (age range 2-57 years). Male to female ration between 1:1 (pediatric cases from Ceará) and 1:8:1 (adult cases from São Paulo). MC subtype predominant in Ceará, while in São Paulo MC and NS cases were equally distributed. Incidence peak at 0-19 years, followed by a descending curve in both States. EBV detected in 64% of the cases, being more common in pediatric cases from Ceará and in adult cases from São Paulo.	15
Vassallo J, et al.	2005	Number of patients: 1,025. Date range (diagnosis): 1990-2000. Pediatric and adult cases (age range not provided) diagnosed in 4 university hospitals and one tertiary cancer center. Male to female ratio: 1.5:1. Incidence peak in the third decade, followed by a descending curve. Predominant subtype: NS. EBV testing: not performed.	16
Araujo I, et al.	2006	Number of patients: 90. Date range (diagnosis): 1976-1993. Pediatric (3-14 years) cases diagnosed in a pediatric hospital in Salvador de Bahia (Northeastern Brazil). Male to female ratio: 3.8:1. EBV detected in 86.7% of the cases, being frequent in all histological subtypes.	17
Chabay PA et al.	2008	Number of patients: 65. Date range (diagnosis): 1998-2003. Pediatric cases (3-18 years) diagnosed in a tertiary cancer care center in the State of Rio de Janeiro (Southeastern Brazil). Male to female ratio: 1.2:1. EBV detectedin 48% of the cases, more common in the MC subtype.	18
Barros MH, et al.	2011	Number of patients: 100. Date range (diagnosis): 1999-2006. Pediatric (up to 18-years old) cases diagnosed in a tertiary cancer center in the State of Rio de Janeiro. Male to female ratio: 1.7:1. Predominant subtype: NS. Number of cases increased with age in direction of the peak typically seen in young adults. EBV detected in 44.8% of the cases. EBV more common in the MC subtype, but detected in approximately 1/3 of the NS cases.	19

3
